# Machine Learning for Lymph Node Metastasis Prediction in Early Gastric Cancer: A Comparative Analysis

**DOI:** 10.7150/ijms.124229

**Published:** 2026-02-11

**Authors:** Yufan Chen, Kunhao Bai, Minghui Yang, Chao Ma, Xiaohang Gao, Guoliang Xu, Yingbo Chen, Rong Zhang

**Affiliations:** 1 Department of Endoscopy, State Key Laboratory of Oncology in South China, Guangdong Provincial Clinical Research Center for Cancer, Sun Yat-sen University Cancer Center, Guangzhou 510060, Guangdong, China.; 2 Department of Pathology, State Key Laboratory of Oncology in South China, Guangdong Provincial Clinical Research Center for Cancer, Sun Yat-sen University Cancer Center, Guangzhou 510060, Guangdong, China.; 3 Department of Gastric Surgery, State Key Laboratory of Oncology in South China, Guangdong Provincial Clinical Research Center for Cancer, Sun Yat-sen University Cancer Center, Guangzhou 510060, Guangdong, China.

**Keywords:** machine learning, gastric cancer, lymph node metastasis, prognosis, prediction model

## Abstract

**Objective:**

Lymph node metastasis (LNM) plays a crucial role in informing treatment decisions and prognosis for early gastric cancer (EGC). This study aimed to offer a practical approach to predict LNM in EGC by using machine learning algorithms.

**Methods:**

This study collected data from 1085 patients with EGC who underwent radical gastrectomy with D1+ or D2 lymph node resection. Seven machine-learning algorithms were compared, and hyperparameters were fine-tuned to identify the model with the best accuracy, Brier class and Area Under the Curve (AUC). The efficacy of the selected model was evaluated.

**Results:**

Following comparison, the Random Forest (RF), Extreme Gradient Boosting (Boost), and Neural Network (NNT) models exhibited exemplary performance on the training dataset, with AUC values of 0.796, 0.788, and 0.779, respectively, on the validation set. We conducted parallel analyses within the T1a and T1b subgroups, where Logistics Models (LM) and RF yielded AUCs of 0.710 and 0.636 in the T1a validation set, and LM, RF, and Boost achieved AUCs of 0.666, 0.658, and 0.558, respectively in the T1b validation set. Variable importance analysis utilizing SHAP revealed distinct values for lymph node metastasis (LNM) in EGC patients, as well as in those stratified into T1a and T1b groups.

**Conclusion:**

The machine learning model holds the potential to guide more effective treatment strategies for early gastric cancer (EGC), specifically in addressing lymph node metastasis (LNM). The identified risk factors contribute valuable insights for personalized decision-making in the management of EGC patients.

## Introduction

Gastric cancer (GC) stands as one of the most frequently diagnosed malignant tumors and ranks as the third leading cause of cancer-related fatalities in China 1,2. Gastric cancer remains a significant global health concern, and the identification of reliable prognostic indicators is crucial for guiding appropriate therapeutic interventions 3. Lymph node metastasis (LNM) plays a crucial role in determining the prognosis and guiding treatment decisions for gastric cancer, especially in the context of early-stage disease 4-6.

Comprehending the clinical and endoscopic features linked to the probability of LNM is essential for crafting effective risk stratification models and refining patient management strategies 7. It assumes a pivotal role in the preliminary assessment of gastric cancer, offering valuable insights into the tumor's biological behavior and the likelihood of metastatic spread. Recognizing specific features associated with an elevated risk of LNM is crucial for refining treatment strategies 8,9.

Several models have been proposed, incorporating factors such as tumor size, depth of invasion, histological type, lymphatic invasion, and molecular markers to predict the probability of lymph node involvement in early gastric cancer. Among these, the nomogram appears to be the most popular, offering a straightforward process yet demonstrating effective predictive capabilities 10-13.

Recently, with advancements in machine learning, more effective methods have been implemented in the field of predicting lymph node involvement in gastric cancer 11,14. Efforts are underway to seamlessly integrate machine learning predictions into the clinical workflow, thereby facilitating real-time decision support for healthcare providers 15,16. Machine learning holds immense promise in revolutionizing the prediction of LNM in EGC. As technology advances and more data becomes available, the collaboration between medical professionals and machine learning experts becomes essential to harness the full potential of these innovative approaches, ultimately improving patient outcomes.

This study aims to delve into the intricate interplay between clinical and endoscopic characteristics and their relationship with the propensity for lymph node involvement in gastric cancer. Through a comprehensive evaluation of nuanced features that may indicate a higher risk of LNM, clinicians can enhance their ability to identify patients who would benefit most from aggressive therapeutic approaches. Moreover, integrating advanced endoscopic techniques allows for a detailed examination of mucosal and submucosal changes, offering an opportunity to refine risk stratification and guide decisions regarding endoscopic resection versus more extensive surgical interventions. The findings of this study hold promise for advancing our understanding of predictive factors, ultimately contributing to the development of more precise risk assessment tools and fostering personalized treatment strategies for patients with gastric cancer.

## Methods

### Study design

We retrieved records of EGC patients who underwent radical gastrectomy for gastric cancer with D1+ or D2 lymph node resection at Sun Yat-Sen Cancer Center (Guangzhou, China) from January 2012 to March 2021. After screening, 1085 records were identified, with pathologically confirmed T1a/T1b stage cases included. Clinical data were extracted from the electronic health records system at Sun Yat-Sen University Cancer Center (SYSUCC).

### Population and definition

Clinicopathological evaluations entailed a comprehensive review of pertinent medical records, specifically blood analysis, gastroscopy, and pathological reports for each participant. Tumor markers, namely A carcinoma embryonic antigen (CEA), CA199, CA125, CA153, and alpha-fetoprotein (AFP), were extracted from the blood analyses. Gastroscopy data, inclusive of tumor localization, were extracted from the respective reports. Pathological outcomes furnished critical information concerning invasion depth (T1a/T1b), histological type, Lauren classification, tumor dimensions, and ulcerative status. The clinical attributes of participants, encompassing gender, age, body mass index (BMI), and personal pathological history, were systematically documented.

The tumors were classified histologically according to the World Health Organization's Classification of Tumors. Differentiated gastric cancer included well-differentiated adenocarcinoma, moderately differentiated adenocarcinoma, and papillary adenocarcinoma. Undifferentiated gastric cancer included poorly differentiated adenocarcinoma, signet-ring cell carcinoma, and mucinous adenocarcinoma 17. The macroscopic types of the tumor were classified according to the Japanese classification of gastric carcinoma. Special pathological types, such as Gastric Fundic Gland Adenocarcinoma, were categorized and summarized under the designation “others.”

The delineated exclusion criteria encompassed 1. patients with antecedent history of neoadjuvant therapy, 2. individuals manifesting two or more primary cancer types, inclusive of gastric and/or other malignancies, 3. patients with antecedent history of cancer or remnant gastric cancer, 4. patients presenting with distant metastasis, and 5. those with incomplete preoperative evaluations (variables demonstrating > 25% information deficit), clinical parameters such as blood analysis, gastroscopy pathological reports, and/or pathological outcomes. These exclusion criteria were systematically applied to the implementation of machine learning (ML) models.

Clinicopathological evaluations entailed a comprehensive review of pertinent medical records, specifically blood analysis, gastroscopy, and pathological reports for each participant. Tumor markers, namely CEA, CA199, CA125, CA153, and AFP, were extracted from the blood analyses. Gastroscopy data, inclusive of tumor localization, were extracted from the respective reports. Pathological outcomes furnished critical information concerning invasion depth (T1a/T1b), histological type, Lauren classification, tumor dimensions, and ulcerative status. The clinical attributes of participants, encompassing gender, age, body mass index (BMI), and personal pathological history, were systematically documented.

### Statistical analysis

Descriptive statistics were employed to summarize the characteristics of the study population. Mean and standard deviation were utilized for continuous variables demonstrating a normal distribution, whereas median and interquartile range (IQR) were employed for non-normally distributed variables. Categorical variables were presented as frequencies and percentages. Two-sided P values less than 0.05 were considered statistically significant. Variables with a p-value less than 0.1 were selected for inclusion in the multivariable analysis.

As not all variables exhibited an effect in predicting LNM, we conducted variable and feature selection using the Boruta method. This method employs an algorithm wrapper built around the random forest classifier and was implemented using the R package Boruta 18. The Boruta method generated a corresponding “shadow” attribute, where values were obtained by shuffling the values of the original attribute across objects, and non-zero values could only result from random fluctuations. Subsequently, the importance of all variables was computed, and the set of importance higher than the shadow was considered confirmed as important, while those lower were rejected. This process aids in distinguishing genuinely important features from those that could arise by chance.

The imbalance in our dataset, with a majority of N0 patients, posed a challenge for model performance, particularly in identifying high-risk LNM positive (N+) patients. To address this, we employed the Synthetic Minority Over-sampling Technique (SMOTE) using the “themis” package in R. Specifically, we utilized the step_smotenc function, setting the over_ratio parameter to 0.25. This approach effectively increased the number of N+ samples to 25% of the N0 sample count, thereby balancing the dataset.

The statistical analyses and machine learning models encompassed association analyses and the application of seven supervised ML classifiers. These classifiers included logistic regression with lasso or elastic net regularization (Logistic), support vector classifier (SVC), extreme gradient boosting (XGBoost), random forest classification (RF), K-Nearest Neighbors (KNN), decision trees (DT), and neural network models (NNET). The models were trained using the aforementioned algorithms, each subjected to a number of tuning parameters, and were subsequently evaluated based on the Receiver Operating Characteristic Area Under the Curve (ROC-AUC). Subsequently, the data was partitioned into a training set and a validation set with a 3:1 ratio. The model with the highest AUC was selected for training on the training set and validation on the validation set. ROC curve, calibration plot, and decision curve analyses were then conducted to evaluate the model. To determine the order of importance in the model, the SHAP (SHapley Additive exPlanations) method 19 was employed to compute the importance score.

The data analysis was conducted using the R language version 4.3.2.

## Results

### The baseline characteristics of the patients

The baseline clinical characteristics of the entire patient cohort are detailed in Table 1, including findings from both univariable and multivariable analyses. Female patients with early-stage gastric cancer showed a higher propensity for lymph node metastasis (LNM), with this trend being statistically significant in both univariable and multivariable analyses (p < 0.001). Patients with LNM (Group N+) tended to be slightly younger than those without metastasis (Group N0), with mean ages of 54±12 years and 56±11 years, respectively. This age difference was evident in the univariable analysis (p=0.006) and was further confirmed in the multivariable analysis (p = 0.007). Although the tumor diameter in the N0 group was smaller compared to the N+ group (2.43±1.96 cm vs. 2.64±1.93 cm, p = 0.022), this difference did not attain statistical significance in the logistic regression analysis. Significant differences were also noted in the macroscopic classification between the two groups, with the 0-IIa type being less likely to exhibit LNM compared to the 0-I type (OR 0.17, 95% CI 0.02 to 0.90). Additionally, CA 199, HLG, pathological type, Lauren classification, T stage, lymphovascular invasion (LVI), and perineural invasion (PI) showed significant differences in the univariable analysis. Of these, the differences in pathological type, T stage, LVI, and PI were further confirmed to be significant in the logistic regression analysis.

Among the 496 patients with T stage 1a disease, 56 had positive lymph nodes. A significant trend was observed among female patients with T stage 1a, who had a higher prevalence of LNM in both univariate (p < 0.001) and multivariate (p = 0.011) analyses. In contrast, patients without LNM were significantly older, with a mean age of 55±12 years compared to 51±12 years (p = 0.012), although this age difference did not remain statistically significant in the multivariate analysis. The impact of pathology type on LNM was also noted, with poorly differentiated gastric cancer being the predominant type associated with LNM in the univariable analysis (p < 0.001), but this association was not sustained in the multivariate analysis (p = 0.19). In a comprehensive analysis, both the Lauren classification and the presence of vascular invasion were identified as independent risk factors for LNM in both univariable and multivariate analyses.

In the analysis of 589 patients with T stage 1b disease, 161 of whom had LNM (Table 3), it was found that female patients were more predominantly affected by LNM in both univariable and multivariable analyses. Younger age emerged (58±11 vs 55±12) as an independent risk factor for LNM in patients in both univariable (p = 0.014) and multivariable analyses (p = 0.029), as did the presence of vascular and nerve invasions. However, despite significant differences in CA199, HLG, and differentiation type in the univariable analysis, the multivariable analysis did not reveal these factors to be statistically significant.

### Variable selection, model comparison, training, and validation of the total cohort

**Figure 1:**
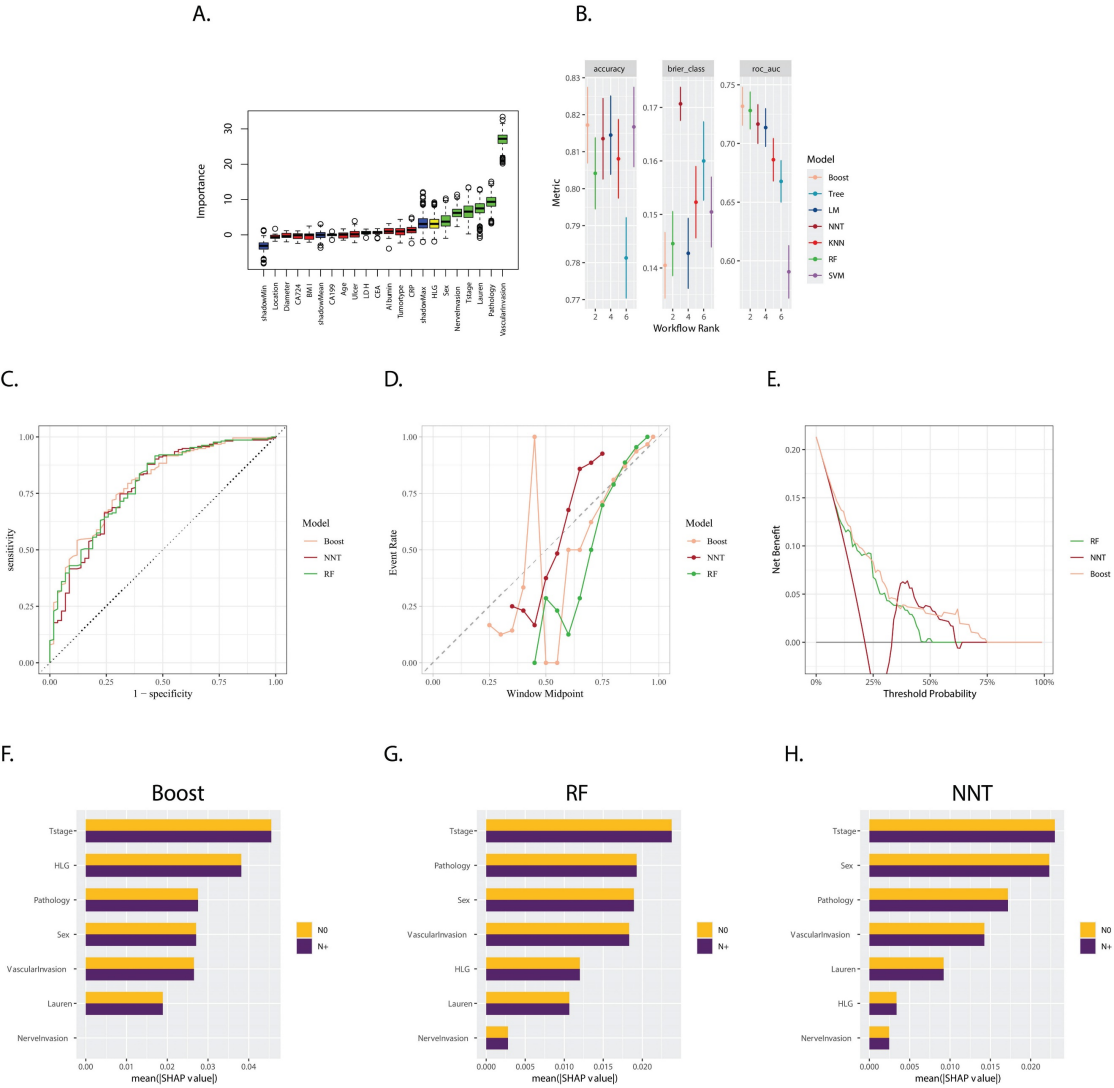
** A.** Boxplot of Variable Importance Selected by Boruta Algorithm: This boxplot displays the importance of variables selected by the Boruta algorithm; **B.** Model Performance in Training Set: The bar chart presents the accuracy, Brier score, and ROC AUC (Area Under the Curve) for seven models trained in the training set. These metrics are used to evaluate the efficacy of the models in predicting the outcome of interest; **C.** ROC Curves for Boost (XGBoost), RF (Random Forest), and NNT (Neural Network) in the validation set: The ROC curves for Boost, RF, and NNT models are shown, with the corresponding AUC values of 0.796, 0.788, and 0.779, respectively; **D.** Calibration Curves for Boost, RF, and NNT: The calibration curves for the three models are presented, which show the agreement between the predicted probabilities and the actual outcomes. A well-calibrated model has predicted probabilities that closely match the observed frequencies; **E.** Decision Curves for Boost, RF, and NNT: The decision curves for the three models are displayed, illustrating the net benefit of using the models at different threshold probabilities. The decision curve analysis helps to assess the clinical utility of the models by considering the trade-off between the benefits and harms of treatment; **F, G, H.** Variable Importance Based on SHAP Values for Boost, RF, and NNT Models: The plots show the variable importance calculated using SHAP (SHapley Additive exPlanations) values for the Boost, RF, and NNT models, respectively. Each bar represents a variable, with the X-axis indicating the SHAP value, which measures the impact of each variable on the model's predictions.

The Boruta feature selection method confirmed the following features as crucial, with “HLG”, “SEX”, “Nerve invasion”, “T stage”, “Lauren type”, “Pathology”, and “Vascular invasion” emerging as the most important in ascending order (Figure 1A). Importantly, these selected variables consistently demonstrated greater significance in each iteration compared to the shadow variables, which are used as a control to assess the importance of the original features.

A multitude of models, incorporating various parameters and machine learning algorithms, were developed using the training datasets. These models were evaluated and ranked based on their accuracy, Brier score, and Area Under the Curve (AUC), as shown in Figure 1B. Notably, three models stood out as the most effective: Extreme Gradient Boosting (Boost), Random Forest (RF), and Neural Network (NNT). The corresponding AUC values for the Boost, RF, and NNT models were 0.796, 0.788, and 0.779, respectively (Figure 1C) in the validation set, indicating considerable predictive performance.

The calibration plot (Figure 1D) revealed that the NNT and RF models exhibited better reliability, suggesting that their predicted probabilities were more closely aligned with the observed outcomes. In contrast, the decision curve analysis (Figure 1E) indicated that the Boost model was more cost-effective across a wider range of threshold probabilities, suggesting its potential clinical utility in decision-making.

The SHapley Additive exPlanations (SHAP) method was employed to compute the importance of variables, as illustrated in Figures 1F, 1G, and 1H. This analysis demonstrated the varying degrees of influence that each variable had on the three models. Although the variables differed in their rankings, “T stage”, “sex”, “pathology type”, “vascular invasion”, and “HLG” consistently emerged as the top variables with significant weight across the models. This highlights their importance in predicting the outcome of interest.

### Variable selection, model comparison, training, and validation of the T stage 1a cohort

**Figure 2:**
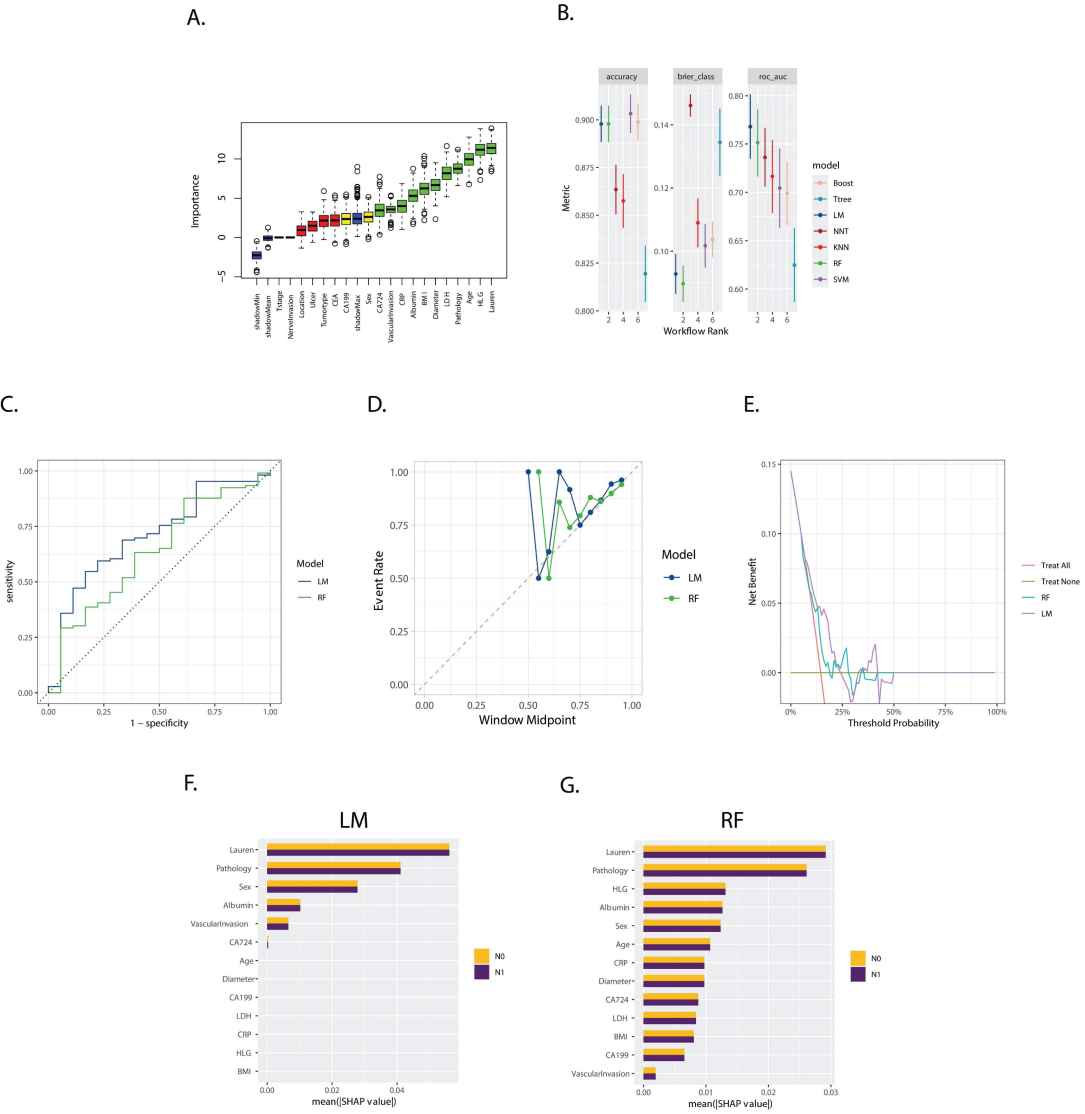
** A.** Boxplot of Variable Importance Selected by Boruta Algorithm: This boxplot displays the importance of variables selected by the Boruta algorithm; **B.** Model Performance in Training Set: The bar chart presents the accuracy, Brier score, and ROC AUC (Area Under the Curve) for seven models trained in the training set. These metrics are used to evaluate the efficacy of the models in predicting the outcome of interest; **C.** ROC Curves for LM (Logistics Models), RF (Random Forest) in the validation set: The ROC curves for LM and RF are shown, with the corresponding AUC values of 0.710, and 0.636, respectively; **D.** Calibration Curves for LM and RF: The calibration curves for the three models are presented, which show the agreement between the predicted probabilities and the actual outcomes. A well-calibrated model has predicted probabilities that closely match the observed frequencies; **E.** Decision Curves for LM and RF: The decision curves for the three models are displayed, illustrating the net benefit of using the models at different threshold probabilities. The decision curve analysis helps to assess the clinical utility of the models by considering the trade-off between the benefits and harms of treatment; **F, G.** Variable Importance Based on SHAP Values for LM and RF models: The plots show the variable importance calculated using SHAP (SHapley Additive exPlanations) values for the LM and RF models, respectively. Each bar represents a variable, with the X-axis indicating the SHAP value, which measures the impact of each variable on the model's predictions.

The Boruta feature selection method identified the following features as crucial, with 'Sex', 'CA724', 'Vascular invasion', 'CRP', 'Albumin', 'BMI', 'Tumor Diameter', 'LDH', 'Pathology', 'Age', 'HLG', and 'Lauren type' emerging as the most important in ascending order (Figure 2A).

Models evaluated and ranked based on accuracy, Brier score, and Area Under the Curve (AUC), as shown in Figure 2B, selected the logistics models (LM) and RF. The corresponding AUC values of which were 0.710 and 0.636, respectively, in the validation set (Figure 2B).

The calibration plot (Figure 2D) revealed the similar reliability of the two models, and the decision curve analysis (Figure 2E) indicated that the two models only worked effectively in a specific range of area to the threshold probability.

The SHapley Additive exPlanations (SHAP) method, illustrated in Figures 2F and 2G, demonstrated “Lauren type”, “Pathology type”, “sex”, “Albumin”, “Vascular invasion”, and “CA 724” as the variables with significant weight across both models, while the RF models considered more variables significantly in the prediction.

### Variable selection, model comparison, training, and validation of the T stage 1b cohort

**Figure 3:**
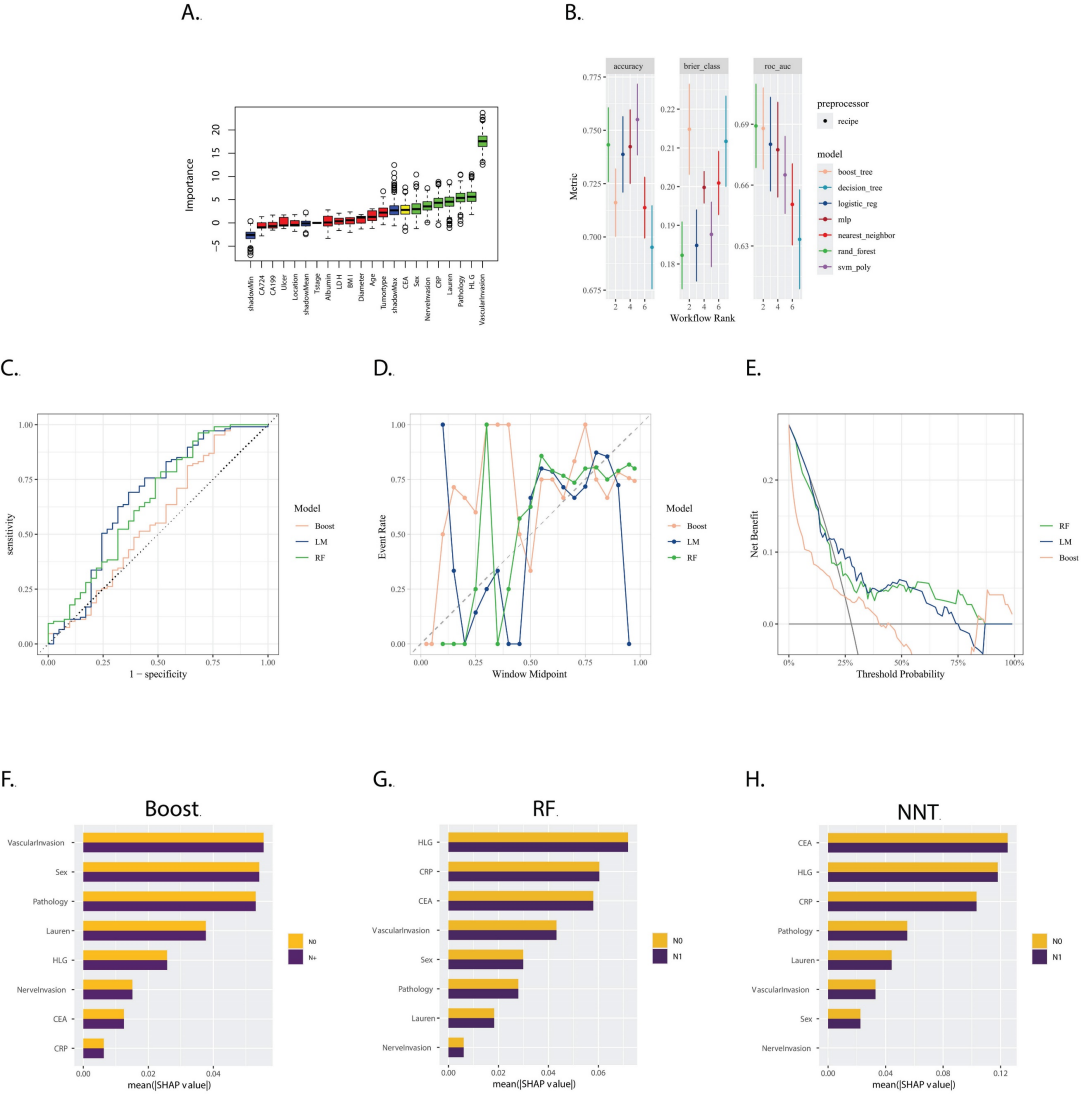
** A.** Boxplot of Variable Importance Selected by Boruta Algorithm: This boxplot displays the importance of variables selected by the Boruta algorithm; **B.** Model Performance in Training Set: The bar chart presents the accuracy, Brier score, and ROC AUC (Area Under the Curve) for seven models trained in the training set. These metrics are used to evaluate the efficacy of the models in predicting the outcome of interest; **C.** ROC Curves for LM, RF and Boost in the validation set: The ROC curves for LM, RF and Boost models are shown, with the corresponding AUC values of 0.666, 0.658 and 0.558, respectively;D. Calibration Curves for LM, RF, and Boost: The calibration curves for the three models are presented, which show the agreement between the predicted probabilities and the actual outcomes. A well-calibrated model has predicted probabilities that closely match the observed frequencies; **E.** Decision Curves for LM, RF and Boost: The decision curves for the three models are displayed, illustrating the net benefit of using the models at different threshold probabilities. The decision curve analysis helps to assess the clinical utility of the models by considering the trade-off between the benefits and harms of treatment; **F, G, H.** Variable Importance Based on SHAP Values for Boost, RF, and NNT Models: The plots show the variable importance calculated using SHAP (SHapley Additive exPlanations) values for the Boost, RF, and NNT models, respectively. Each bar represents a variable, with the X-axis indicating the SHAP value, which measures the impact of each variable on the model's predictions.

The Boruta feature selection method identified 'CEA', 'Sex', 'Nerve invasion', 'CRP', 'Lauren type', 'Pathology', 'HLG', and 'Vascular invasion' as the most crucial features for predicting gastric cancer prognosis, ranked in ascending order of importance (Figure 3A).

Models were evaluated and ranked based on accuracy, Brier score, and Area Under the Curve (AUC). As depicted in Figure 3B, the Random Forest (RF), Boosting, and Logistics Model (LM) were selected. Their corresponding AUC values in the validation set were 0.658 for RF, 0.558 for Boosting, and 0.666 for LM (Figure 3B).

The calibration plot (Figure 3D) revealed that the reliability of the three models was similar, with none performing exceptionally well. The decision curve analysis (Figure 3E) indicated that the RF model was more effective over a wider range of threshold probabilities.

The SHapley Additive exPlanations (SHAP) method, illustrated in Figures 3F, 3G, and 3H, demonstrated that the weight of the variables was quite different between the LM and the RF and Boost models. This suggests that each model considered different factors or interactions among factors in making predictions.

## Discussion

Early gastric cancer is linked to the risk of lymph node metastasis (LNM), a crucial factor in determining prognosis and guiding treatment strategies. Various clinical and pathological factors play a role in evaluating the probability of lymph node involvement in cases of early gastric cancer. Based on our findings in the current study, sex, age, macroscopic type, tumor depth, lympho-vascular invasion (LVI), and perineural invasion (PI) demonstrated significance in both univariable and multivariable analyses. The results confirmed that these factors are independent risk factors for lymph node metastasis (LNM) in patients with early gastric cancer (EGA). In our study, it was observed that female patients encountered greater challenges related to lymph node metastasis, consistent with previous reports 20,21. This underscores clinicians' importance in prioritizing female patients and considering adopting a potentially more aggressive treatment strategy.

Additionally, for the whole cohort, our analysis identified age as an independent risk factor, with younger patients appearing to have a higher risk of lymph node metastasis (LNM), aligning with findings from previous literature 22. This observation can be attributed to the higher likelihood of younger patients having aggressive tumor characteristics. Specifically, gastric cancer in younger individuals often presents with poorly differentiated tumors and is commonly of the mixed or diffuse Lauren type, in contrast to the more differentiated tumors and Lauren intestinal type typically observed in older patients 23,24. Furthermore, the macroscopic type, as for the Japanese classification of gastric carcinoma, emerged as an independent risk factor, indicating its close association with prognosis and, consequently, its potential influence on treatment strategy 25. Tumor depth exhibited a significant correlation with lymph node metastasis (LNM) 26. As the tumor progresses in depth, there is an increased likelihood of breaching the lymphatic vessels, facilitating the dissemination of cancer cells to regional lymph nodes. Lymphovascular invasion (LVI) and perineural invasion (PI) were established as independent risk factors through various analyses 8,10,27. The lymphatic vessels act as a conduit for cancer cells to migrate from the primary tumor site to adjacent lymph nodes, providing these cells with entry into the lymphatic system and facilitating their dissemination to regional lymph nodes. The presence of lymphovascular invasion (LVI) indicates a more aggressive tumor behavior and a heightened risk of systemic spread. Perineural invasion (PI) entails the infiltration of cancer cells into the perineural space, enabling them to utilize nerve fibers as a pathway for local spread 28. When cancer cells invade nerves, they may follow nerve pathways to reach nearby lymph nodes. PI is often regarded as a marker of more aggressive tumor behavior. Conversely, the microenvironment surrounding nerves contains neurotrophic factors that may attract and support the growth of cancer cells.

In the T1a group, historically regarded as having a low probability of lymph node metastasis (LNM), the observed LNM rate was 11.2% (56 out of 496 cases), challenging the previous understanding. The model demonstrated robust performance in the validation dataset. Utilizing SHapley Additive exPlanations (SHAP), we elucidated the substantial impact of Lauren classification and pathology on both the logistic regression (LM) and random forest (RF) models. Multivariable analysis indicated an odds ratio of 4.91 (95% confidence interval: 1.51, 19.9) for mixed-type tumors versus intestinal-type tumors, affirming the elevated risk associated with mixed-type tumors, in line with previous findings 29, Similarly, poorly differentiated carcinomas also exhibited an increased risk, 30. Lymphovascular invasion (LVI) was observed infrequently in the T1a group (2 out of 496 cases), reflecting a low incidence of LVI in patients with cancer limited to the mucosal layer. Notably, both T1a patients with positive LVI were subsequently confirmed to have lymph node metastasis (N+) following surgery. This finding underscores LVI as a potent risk factor for T1a patients. Despite its clinical significance, LVI did not carry substantial weight in the SHAP analysis, revealing a discrepancy between its actual risk and the model's representation. A technical explanation for the discrepancy between the clinical significance of Lymphovascular Invasion (LVI) and its low importance in the SHAP analysis was due to the limited number of LVI+ cases, which may not provide enough examples for the model to learn the pattern associated with LVI as a strong predictor of LNM. Additionally, SHAP values were based on the average marginal contribution of a feature, which may not fully capture the impact of rare events like LVI. This affected the model's reliability in guiding critical decisions, such as refining risk stratification for endoscopic resection versus surgical intervention. Clinicians should be cautious when interpreting the model's predictions, especially in cases involving rare events with strong clinical significance. Further research is needed to develop models that can better handle rare events and their clinical significance.

In the T1b subgroup, the observed lymph node metastasis (LNM) rate was 27.3% (161 out of 589 cases). The models developed for this group demonstrated less efficacy compared to those for the T1a group and the overall cohort. There was considerable variation in the rankings of variable importance among the boosting, logistic regression (LM), and random forest (RF) models. Notably, while lymphovascular invasion (LVI) and perineural invasion (PI) were both identified as independent risk factors in both univariate and multivariate analyses, the relative importance of LVI varied across the different models. Furthermore, PI was not consistently ranked as a high-importance variable across all models.

The observed lack of efficacy in the T1b subgroup models, as compared to the T1a group and overall cohort models, may be attributed to several factors. Machine learning methods provide predictive model weights from a computational perspective. However, due to limitations such as sample size constraints and data heterogeneity, variables that are clinically considered more relevant may not exert as significant an influence on model predictions as those deemed less relevant. This underscores the need for further validation of the selected model variables through molecular biology.

The variability in variable importance rankings, particularly for lymphovascular invasion (LVI) and perineural invasion (PI), across different models in the T1b subgroup suggests that these factors may have different levels of impact on LNM in this specific subgroup. This variability could stem from the inherent complexity and heterogeneity of the T1b subgroup, as well as the differences in model algorithms and feature selection methods used. While LVI and PI are highly correlated with LNM risk, as confirmed in both univariate and multivariate analyses, their weights in the predictive model may be lower due to sample size and data heterogeneity. Therefore, further research is necessary to identify more clinically valuable predictive variables.

The advancements in machine learning have led to the development of more accurate and efficient models for diagnosing specific clinical issues. Although its application has been predominantly in the fields of omics and artificial intelligence 31, it has also demonstrated promising efficacy in traditional clinical research 32. Particularly, the application of explainable machine learning allows us to understand the importance of various variables within a model, enabling a better comprehension of the relationship between variables and phenotypes.

Boruta is a feature selection method employed in machine learning, specifically designed to identify and choose crucial features within a dataset by comparing them to shadow attributes 33. This method proves valuable when dealing with high-dimensional datasets, where not all features significantly contribute to the model's performance. Utilizing a random forest classifier, Boruta is a non-linear approach capable of capturing complex relationships and interactions between features, aspects that may be overlooked by linear methods such as Lasso Regression. One notable strength of Boruta lies in its enhanced robustness to multicollinearity, a condition of high correlation between features. In contrast, Lasso Regression may arbitrarily select one variable over another in the presence of high correlation, potentially leading to instability in variable selection. Additionally, Boruta adopts a model-agnostic approach, refraining from assuming a specific form of the relationship between features and the target variable. This flexibility enables its application across various types of models.

It is crucial to acknowledge that while Boruta is a powerful tool, it may exhibit overfitting tendencies on the training data. Multiple iterations were employed to thoroughly assess its effectiveness in the present study to avoid this problem.

As the machine learning algorithm demonstrated dataset agnosticism, the performance of a specific model could vary across different scenarios. To address this, we systematically evaluated the performance of various algorithms and fine-tuned the hyperparameters of selected models. The goal was to identify the model with the highest Area Under the Curve (AUC) during the ROC curve analysis.

Although the ROC curve analysis indicated satisfactory performance of these models on the test set, further examination through calibration plots revealed discrepancies, with several predictions deviating from the observed outcomes in both the training and test sets. This discrepancy may be attributed to the fact that the sensitivities were relatively low while specificities were high.

An analysis of the imbalance in the dataset sheds light on a potential cause for the discrepancy. The majority of the dataset comprised N0 patients, leading the model to adopt a strategy that predicted more patients in the N0 group. This strategy aimed to enhance the AUC, implying that the model prioritized correctly predicting the majority of N0 patients. However, this strategy might have contributed to lower sensitivities, impacting the overall predictive accuracy. The computed importance of variables by the Boruta method and the SHA methods exhibited notable differences. SHAP is a model-agnostic algorithm used to explain the output of any machine learning model. It assigns each feature an importance value for a particular prediction, which helps in understanding the model's decision-making process.

Although machine learning models are frequently regarded as opaque, the integration of SHAP offers a crucial layer of interpretability. This feature is especially vital in clinical settings, where it assists in pinpointing which risk factors require heightened focus. By comprehending these subtleties, healthcare professionals can concentrate on pivotal variables and make well-informed decisions informed by the model's predictions.

Nevertheless, our research successfully developed effective models that prioritized risk factors significantly correlated with lymph node metastasis in patients with early gastric cancer (EGC), encompassing stages T1a, T1b, and the entire cohort. This advancement offers clinicians invaluable insights, enhancing their understanding of the determinants of lymph node metastasis and assisting in the formulation of targeted treatment strategies.

## Conclusion

The machine learning model holds the potential to guide more effective treatment strategies for early gastric cancer (EGC), specifically in addressing lymph node metastasis (LNM). The identified risk factors contribute valuable insights for personalized decision-making in the management of EGC patients.

## Figures and Tables

**Table 1:** Baseline characteristics of the total patients with both T stage 1a and 1b.

	Univariable			Multivariable		
Characteristic	N0, N = 868^1^	N+, N = 217^1^	p-value^2^	OR^3^	95% CI^3^	p-value
**Sex**			**< 0.001**			**< 0.001**
F	304 (35%)	116 (53%)		—	—	
M	564 (65%)	101 (47%)		0.43	0.30, 0.63	
**Age**	56±11	54±12	**0.006**	0.98	0.96, 0.99	**0.007**
**Diameter**	2.43±1.96	2.64±1.93	**0.022**	1.05	0.97, 1.14	0.20
**Tumor type**			**0.012**			**0.043**
0-I	20 (2.3%)	8 (3.7%)		—	—	
0-IIa	34 (3.9%)	2 (0.9%)		0.17	0.02, 0.90	
0-IIb	185 (21%)	34 (16%)		0.48	0.18, 1.37	
0-IIc	4 (0.5%)	0 (0%)		0.00		
0-III	34 (3.9%)	5 (2.3%)		0.51	0.10, 2.35	
Borrmann I	60 (6.9%)	15 (6.9%)		0.64	0.21, 2.02	
Borrmann II	444 (51%)	116 (53%)		1.28	0.28, 5.74	
Borrmann III	70 (8.1%)	32 (15%)		2.35	0.48, 11.4	
Borrmann IV	17 (2.0%)	5 (2.3%)		0.36	0.07, 1.64	
**Ulcer**			0.063			0.32
No	331 (38%)	68 (31%)		—	—	
Ulcer	537 (62%)	149 (69%)		0.54	0.17, 1.85	
**Location**			0.7			
L	549 (63%)	131 (60%)				
M	244 (28%)	67 (31%)				
U	75 (8.6%)	19 (8.8%)				
**CEA**	2.47±3.22	2.83±6.04	0.090	1.02	0.99, 1.06	0.20
**CA199**	26±216	38±227	**0.037**	1.00	1.00, 1.00	0.82
**CA724**	3.12±9.78	3.55±7.10	0.7			
**LDH**	162±30	164±36	> 0.9			
**Albumin**	43.7±19.2	42.5±3.7	0.10	0.97	0.92, 1.00	0.11
**CRP**	4.8±38.4	13.0±137.0	0.3			
**HLG**	134±21	129±20	**< 0.001**	1.00	0.99, 1.01	0.82
**Pathology**			**< 0.001**			**0.043**
Poor	589 (68%)	177 (82%)		—	—	
Special	15 (1.7%)	7 (3.2%)		1.78	0.61, 4.79	
Well	264 (30%)	33 (15%)		0.53	0.30, 0.92	
**Lauren**			**< 0.001**			0.15
Intestinal	363 (42%)	66 (30%)		—	—	
Mix	187 (22%)	73 (34%)		1.18	0.70, 1.98	
Diffuse	318 (37%)	78 (36%)		0.78	0.46, 1.32	
**T stage**			**< 0.001**			**< 0.001**
T1a	440 (51%)	56 (26%)		—	—	
T1b	428 (49%)	161 (74%)		2.44	1.70, 3.54	
**Vascular Invasion**			**< 0.001**			**< 0.001**
Present	13 (1.5%)	37 (17%)		—	—	
Absent	855 (99%)	180 (83%)		0.12	0.06, 0.24	
**Nerve Invasion**			**< 0.001**			**0.025**
Present	6 (0.7%)	9 (4.1%)		—	—	
Absent	862 (99%)	208 (96%)		0.24	0.07, 0.83	
**BMI**	22.6±13.1	23.7±31.8	0.4			

^1^n (%); Mean±SD; ^2^Pearson's Chi-squared test; Wilcoxon rank sum test; Fisher's exact test; ^3^OR = Odds Ratio, CI = Confidence Interval

**Table 2:** Baseline characteristics of the patients with T stage of 1a.

	Univariable				Multivariable		
**Characteristic**	**Overall**, N = 496^1^	**0**, N = 440^1^	**1**, N = 56^1^	**p-value** ^2^	**OR** ^3^	**95% CI** ^3^	**p-value**
**Sex**				**<0.001**			**0.011**
F	214 (43%)	178 (40%)	36 (64%)		—	—	
M	282 (57%)	262 (60%)	20 (36%)		0.38	0.17, 0.80	
**Age**	55±12	55±12	51±12	**0.012**	0.98	0.95, 1.01	0.24
**Diameter**	2.34±1.55	2.33±1.60	2.43±1.09	0.12	0.97	0.76, 1.21	0.79
**Tumor type**				0.22			0.067
0-I	14 (2.8%)	13 (3.0%)	1 (1.8%)		—	—	
0-IIa	26 (5.2%)	26 (5.9%)	0 (0%)		0.00	NA	
0-IIb	124 (25%)	109 (25%)	15 (27%)		2.49	0.37, 50.2	
0-IIc	3 (0.6%)	3 (0.7%)	0 (0%)		0.00	NA	
0-III	25 (5.0%)	23 (5.2%)	2 (3.6%)		0.71	0.02, 25.4	
Borrmann I	25 (5.0%)	24 (5.5%)	1 (1.8%)		1.07	0.04, 31.5	
Borrmann II	226 (46%)	198 (45%)	28 (50%)		2.10	0.09, 68.9	
Borrmann III	42 (8.5%)	33 (7.5%)	9 (16%)		4.89	0.18, 179	
Borrmann IV	11 (2.2%)	11 (2.5%)	0 (0%)		0.00	NA	
**Ulcer**				0.089			0.78
No	212 (43%)	194 (44%)	18 (32%)		—	—	
Ulcer	284 (57%)	246 (56%)	38 (68%)		1.36	0.18, 19.4	
**Location**				0.6			
L	335 (68%)	297 (68%)	38 (68%)				
M	138 (28%)	121 (28%)	17 (30%)				
U	23 (4.6%)	22 (5.0%)	1 (1.8%)				
**CEA**	2.40±3.71	2.46±3.91	1.88±1.32	0.054	0.93	0.70, 1.14	0.55
**CA199**	25±202	27±215	13±9	0.6	0.99	0.96, 1.00	0.40
**CA724**	3.13±8.96	3.15±9.40	2.99±4.27	0.7			
**LDH**	162±31	162±30	163±38	0.9			
**Albumin**	42.9±3.2	43.0±3.3	42.6±2.5	0.2	0.94	0.85, 1.05	0.30
**CRP**	9.24±102.76	5.45±51.98	39.07±269.15	0.9			
**HLG**	133±20	133±20	130±15	0.053	1.00	0.98, 1.02	0.79
**Pathology**				**< 0.001**			0.19
Poor	345 (70%)	296 (67%)	49 (88%)		—	—	
Special	11 (2.2%)	8 (1.8%)	3 (5.4%)		2.41	0.47, 9.70	
Well	140 (28%)	136 (31%)	4 (7.1%)		0.34	0.06, 1.50	
**Lauren**				**< 0.001**			**< 0.001**
Intestinal	175 (35%)	169 (38%)	6 (11%)		—	—	
Mix	100 (20%)	74 (17%)	26 (46%)		4.91	1.51, 19.9	
Diffuse	221 (45%)	197 (45%)	24 (43%)		1.34	0.40, 5.61	
**Vascular Invasion**				**0.013**			**< 0.001**
Present	2 (0.4%)	0 (0%)	2 (3.6%)		—	—	
Absent	494 (100%)	440 (100%)	54 (96%)		0.00		
**Nerve Invasion**				> 0.9			0.57
Present	1 (0.2%)	1 (0.2%)	0 (0%)		—	—	
Absent	495 (100%)	439 (100%)	56 (100%)		20,442,763	0.00, NA	
**BMI**	21.75±6.39	21.78±6.71	21.47±3.01	0.5			

^1^n (%); Mean±SD; ^2^Pearson's Chi-squared test; Wilcoxon rank sum test; Fisher's exact test; OR = Odds Ratio, CI = Confidence Interval

**Table 3:** Baseline characteristics of the patients with T stage of 1b.

	Univariable				Multivariable		
Characteristic	Overall, N = 589^1^	0, N = 428^1^	1, N = 161^1^	p-value^2^	OR^3^	95% CI^3^	p-value
**Sex**				**< 0.001**			**< 0.001**
F	206 (35%)	126 (29%)	80 (50%)		—	—	
M	383 (65%)	302 (71%)	81 (50%)		0.44	0.28, 0.68	
**Age**	57±11	58±11	55±12	**0.014**	0.98	0.96, 1.00	**0.029**
**Diameter**	2.58±2.24	2.53±2.27	2.72±2.15	0.2	1.06	0.97, 1.16	0.17
**Tumor type**				0.11			0.12
0-I	14 (2.4%)	7 (1.6%)	7 (4.3%)		—	—	
0-IIa	10 (1.7%)	8 (1.9%)	2 (1.2%)		0.21	0.02, 1.58	
0-IIb	95 (16%)	76 (18%)	19 (12%)		0.22	0.06, 0.81	
0-IIc	1 (0.2%)	1 (0.2%)	0 (0%)		0.00		
0-III	14 (2.4%)	11 (2.6%)	3 (1.9%)		0.47	0.05, 3.39	
Borrmann I	50 (8.5%)	36 (8.4%)	14 (8.7%)		0.46	0.12, 1.77	
Borrmann II	334 (57%)	246 (57%)	88 (55%)		1.33	0.17, 10.5	
Borrmann III	60 (10%)	37 (8.6%)	23 (14%)		2.26	0.27, 19.3	
Borrmann IV	11 (1.9%)	6 (1.4%)	5 (3.1%)		0.26	0.04, 1.70	
**Ulcer**				0.8			0.19
No	187 (32%)	137 (32%)	50 (31%)		—	—	
Ulcer	402 (68%)	291 (68%)	111 (69%)		0.32	0.06, 1.74	
**Location**				0.8			
L	345 (59%)	252 (59%)	93 (58%)				
M	173 (29%)	123 (29%)	50 (31%)				
U	71 (12%)	53 (12%)	18 (11%)				
**CEA**	2.67±4.13	2.48±2.31	3.17±6.94	0.2	1.05	1.0, 1.12	0.083
**CA199**	31±231	25±217	46±263	**0.028**	1.00	1.00, 1.00	0.80
**CA724**	3.3±9.6	3.1±10.2	3.7±7.9	0.5			
**LDH**	164±32	163±31	164±35	0.9			
**Albumin**	44.0±23.3	44.5±27.2	42.4±4.0	0.3	0.98	0.93, 1.00	0.24
**CRP**	4.1±13.6	4.1±14.7	4.0±9.9	0.5			
**HLG**	133±22	134±22	129±21	**0.002**	1.00	0.99, 1.01	0.74
**Pathology**				**0.009**			0.17
Poor	421 (71%)	293 (68%)	128 (80%)		—	—	
Special	11 (1.9%)	7 (1.6%)	4 (2.5%)		1.39	0.31, 5.54	
Well	157 (27%)	128 (30%)	29 (18%)		0.56	0.30, 1.04	
**Lauren**				0.2			0.64
Intestinal	254 (43%)	194 (45%)	60 (37%)		—	—	
Mix	160 (27%)	113 (26%)	47 (29%)		0.77	0.42, 1.40	
Diffuse	175 (30%)	121 (28%)	54 (34%)		0.77	0.42, 1.42	
**Vascular Invasion**				**< 0.001**			**< 0.001**
Present	48 (8.1%)	13 (3.0%)	35 (22%)		—	—	
Absent	541 (92%)	415 (97%)	126 (78%)		0.15	0.07, 0.30	
**Nerve Invasion**				**0.004**			**0.013**
Present	14 (2.4%)	5 (1.2%)	9 (5.6%)		—	—	
Absent	575 (98%)	423 (99%)	152 (94%)		0.20	0.05, 0.71	
**BMI**	23.8±24.2	23.5±17.3	24.5±36.8	0.3			

^1^n (%); Mean±SD; ^2^Pearson's Chi-squared test; Wilcoxon rank sum test; Fisher's exact test; ^3^OR = Odds Ratio, CI = Confidence Interval

## Data Availability

The data that support the findings of this study are available on request from the corresponding author. The data are not publicly available due to privacy or ethical restrictions.

## Abbreviations

AFP: alpha-fetoprotein
AUC: Area Under the Curve
BMI: body mass index
CEA: carcinoma embryonic antigen
CI: Confidence Interval
DT: decision trees
EGC: early gastric cancer
XGBoost: extreme gradient boosting
Boost: Extreme Gradient Boosting
GC: Gastric cancer
IQR: interquartile range
KNN: K-Nearest Neighbors
LM: Logistics Models
LNM: lymph node metastasis
LVI: lymphovascular invasion
ML: machine learning
NNT: Neural Network
NNET: neural network models
OR: Odds Ratio
PI: perineural invasion
RF: Random Forest
ROC-AUC: Receiver Operating Characteristic Area Under the Curve
SHAP: SHapley Additive exPlanations
SVC: support vector classifier
